# Proteome Analysis for Inflammation Related to Acute and Convalescent Infection

**DOI:** 10.1007/s10753-023-01913-3

**Published:** 2023-10-13

**Authors:** Tara K. Sigdel, Swastika Sur, Patrick Boada, Suzanne M. McDermott, Cecilia S. Lindestam Arlehamn, Kristy O. Murray, Linda K. Bockenstedt, Maggie Kerwin, Elaine F. Reed, Eva Harris, Ken Stuart, Bjoern Peters, Ana Sesma, Ruth R. Montgomery, Minnie M. Sarwal

**Affiliations:** 1 Division of Multi-Organ Transplantation, Department of Surgery, University of California San Francisco, 513 Parnassus Ave, Med Sciences Bldg, Room S1268, San Francisco, CA 94143 USA; 2 Seattle Children Research Institute, Seattle, WA USA; 3 Department of Pediatrics, University of Washington School of Medicine, Seattle, WA USA; 4 La Jolla Institute for Immunology, La Jolla, CA USA; 5 Baylor College of Medicine, Houston, TX USA; 6 Yale School of Medicine, New Haven, CT USA; 7 Department of Pathology and Laboratory Medicine, David Geffen School of Medicine, University of California Los Angeles, Los Angeles, CA USA; 8 University of California Berkeley, Berkeley, CA USA; 9 Mount Sinai School of Medicine, New York, NY USA

**Keywords:** Pathogens, Proteomics, BK virus, CMV, Tuberculosis, Malaria, West Nile virus, Lyme disease, Dengue

## Abstract

Infectious diseases are a significant burden in global healthcare. Pathogens engage with different host defense mechanisms. However, it is currently unknown if there are disease-specific immune signatures and/or if different pathogens elicit common immune-associated molecular entities to common therapeutic interventions. We studied patients enrolled through the Human Immunology Project Consortium (HIPC), which focuses on immune responses to various infections. Blood samples were collected and analyzed from patients during infection and follow-up time points at the convalescent stage. The study included samples from patients with Lyme disease (LD), tuberculosis (TB), malaria (MLA), dengue virus (DENV), and West Nile virus (WNV), as well as kidney transplant patients with cytomegalovirus (CMV) and polyomavirus (BKV) infections. Using an antibody-based assay, we quantified ~ 350 cell surface markers, cytokines, and chemokines involved in inflammation and immunity. Unique protein signatures were identified specific to the acute phase of infection irrespective of the pathogen type, with significant changes during convalescence. In addition, tumor necrosis factor receptor superfamily member 6 (TNR6), C–C Motif Chemokine Receptor 7 (CCR7), and C–C motif chemokine ligand-1 (CCL1) were increased in the acute and convalescent phases across all viral, bacterial, and protozoan compared to blood from healthy donors. Furthermore, despite the differences between pathogens, proteins were enriched in common biological pathways such as cell surface receptor signaling pathway and response to external stimulus. In conclusion, we demonstrated that irrespective of the pathogen type, there are common immunoregulatory and proinflammatory signals.

**Supplementary Information:**

The online version contains supplementary material available at 10.1007/s10753-023-01913-3.

## INTRODUCTION

Infectious diseases caused by communicable pathogens including viruses, bacteria, fungi, and parasites are listed by the World Health Organization as among the leading causes of death worldwide [1]. The host–pathogen dyad is a dynamic system, as the virulence factors and host responses are in constant flux during the course of the infection [2]. Pathogens have evolved numerous strategies to overcome innate and adaptive immunity and escape host immune elimination. This balance of host immune response and pathogen counter-defense contributes to the complexity and diversity of pathological infections [3].

Studying host response toward multiple pathogens has proven useful in determining unique and universal elements of host defense mechanisms [4]. In a recent report, we studied two common arthropod-borne infections: Lyme disease (LD), an extracellular bacterial infection, and West Nile virus infection (WNV), an intracellular viral infection and showed similarities in host responses despite the different vectors and the pathogens [5]. Understanding acute and recovery phase immune signatures of the host when exposed to a variety of broad pathogen families (bacteria, viruses, and protozoa) can provide a greater understanding of host defense and pathogen clearance or persistence mechanisms. Pathogens invade, survive, replicate, and colonize through diverse mechanisms involving both cellular and humoral immunity and with the engagement of both innate and adaptive immunity [3]. These pathogens can exist in circulation or reside in the intracellular niches, and they encounter attacks that include those by host cytotoxic T lymphocytes, macrophage and monocyte systems, natural killer (NK) cells, NK T cells, γδ T cells, cytotoxic cytokines, antibodies, and complement cascade [6–8]. Infection with mycobacteria, spirochetes, members of the Herpesviridae family, and fungal infections elicit macrophage hyperactivation and granuloma formation, which localize or clear the infection [9]. Once an infection is established, its clearance or persistence in tissue and cellular niches is interconnected to a balance of host immunity, host genetics, pathogen virulence, pathogen mutations, and other innate survival mechanisms [10, 11]. Outcomes are determined by factors related to both the host and pathogen and the extent of host–pathogen interactions.

We hypothesized that phylogenetically diverse microbes (bacterial, viral, protozoal) might share common pathogen subtype-specific host–pathogen interactions and host defense mechanisms while also eliciting unique host immune signatures specific to an individual pathogen. We also predicted that commonalities of immune and molecular profiles might exist across the acute and chronic/recovery phases of infections, regardless of inherent variations across pathogen species. We have conducted a pilot proteomic study on paired blood samples collected from patients, with Lyme disease (LD), tuberculosis (TB), malaria (MLA), cytomegalovirus (CMV), polyomaviridae (BKV), dengue virus (DENV), and West Nile virus (WNV), during infection and later in the convalescent stage. This study tracks the pathways that are crucial for intracellular pathology (all pathogens are intracellular except for *Borrelia burgdorferi* that causes LD), microbial defense, and the associated post-infection changes in the host. Our proteomic analyses of diverse infection-induced signatures provide a unique opportunity to identify meta-signatures that characterize host–pathogen interactions, inflammatory responses, and the post-inflammatory milieu.

Our study design incorporates longitudinal, paired samples from these diverse patients within each infection group with different infections and interrogates immune pathways that support clearance of a range of pathogens over time. The diversity of infections allows us to identify both common and unique immune pathways. This study improves our understanding of host–pathogen dynamics that could aid in the development of vital disease diagnostics, interventions, and vaccines that are more needed then ever given the global increase in the incidence of emerging and reemerging infectious diseases.

## RESULTS

**Fig. 1:**
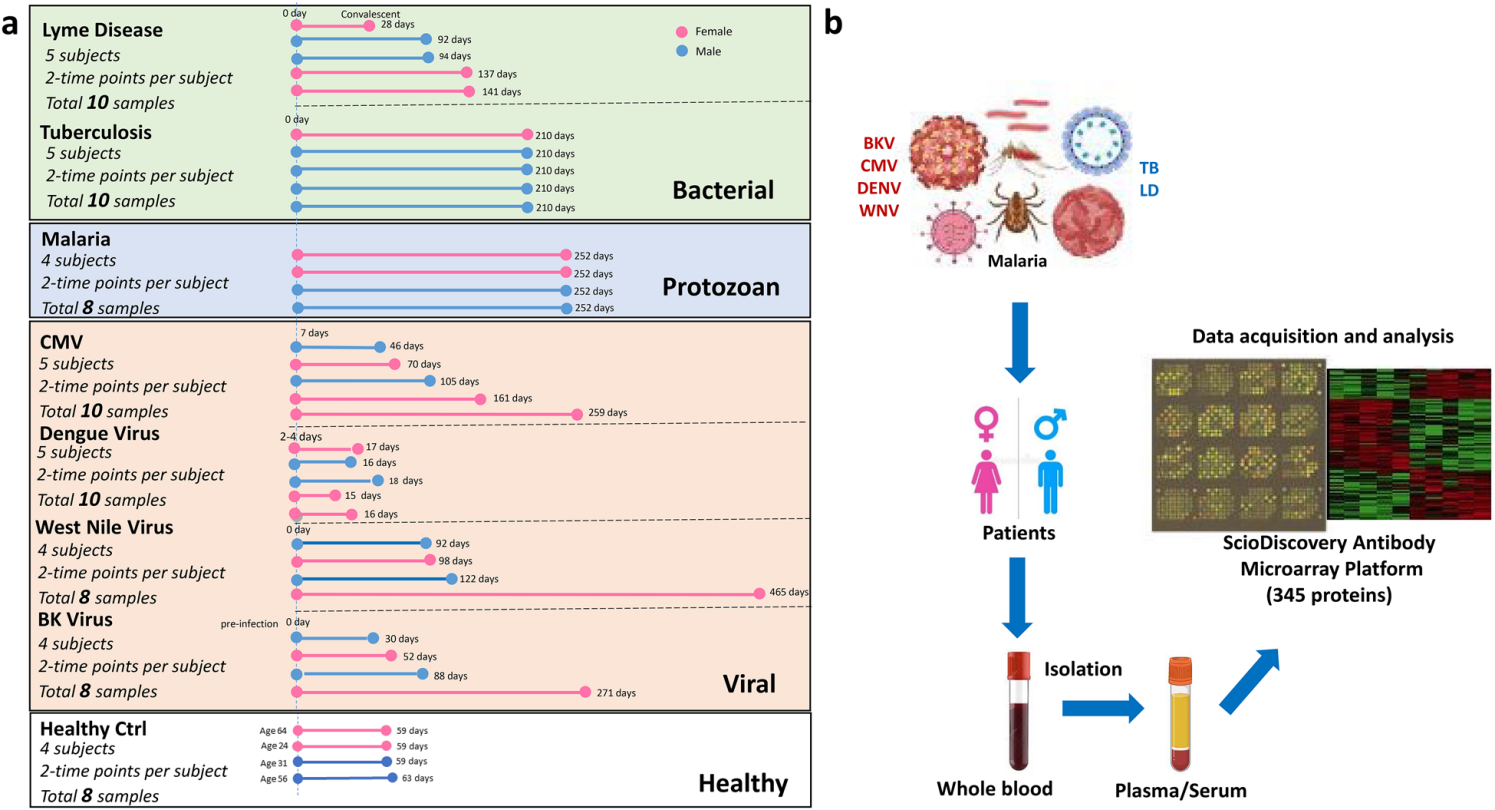
Scheme illustrating specimen collection per infection and protein expression analysis platform. **a** Barplot demonstrating the dispersion of sample-collection timepoints, gender, and disease state across seven distinct diseases (BKV, cytomegalovirus, dengue, Lyme disease, malaria, tuberculosis, and West Nile virus) and healthy controls. **b** Overview of the infections studied, specimens collected, and platform used for the data acquisition. scioCD antibody microarray platform profiled the expression of 345 proteins at any given time in plasma or serum isolated from blood.

Characteristics of Patients with Bacterial (LD and TB), Protozoan (MLA), and Viral (CMV, BKV, DENV, WNV) Infection. Patients’ age, gender, and the time points of sample collection across seven distinct infections and overall study schematic are presented in Fig. 1 and Table 1. Based on the types of pathogen and diseases, we used either serum (LD, WNV) or plasma (CMV, DENV, TB, BKV, and MLA) isolated from patients’ blood with each infection for the scioCD antibody microarray assay. Blood samples collected 1 month apart from healthy adult individuals (HC) were used as controls. Also, patients with CMV and BKV have undergone kidney transplantation. Samples were collected at two different time points, one at the time of infection or acute phase and the other at the convalescent/recovery phase, the time at which the patients were checked for signs of recovery after they were diagnosed with the disease in the acute phase.

**Table 1:** Summary Table for Study Subjects and Samples

Cohort name	Total patients (male/female)	Total number of samples	Blood sample
Lyme disease (LD)	5 (2/3)	10	Serum
Tuberculosis	5 (4/1)	10	Plasma
Malaria	4 (2/2)	8	Plasma
Cytomegalovirus (CMV)	5 (2/3)	10	Plasma
Dengue virus	5 (2/3)	10	Plasma
West Nile virus	4 (2/2)	8	Serum
BK virus (BKV)	4 (2/2)	8	Plasma
Healthy controls	4 (2/2)	8	Plasma
Total	36 (18/18)	72	Plasma

**Fig. 2:**
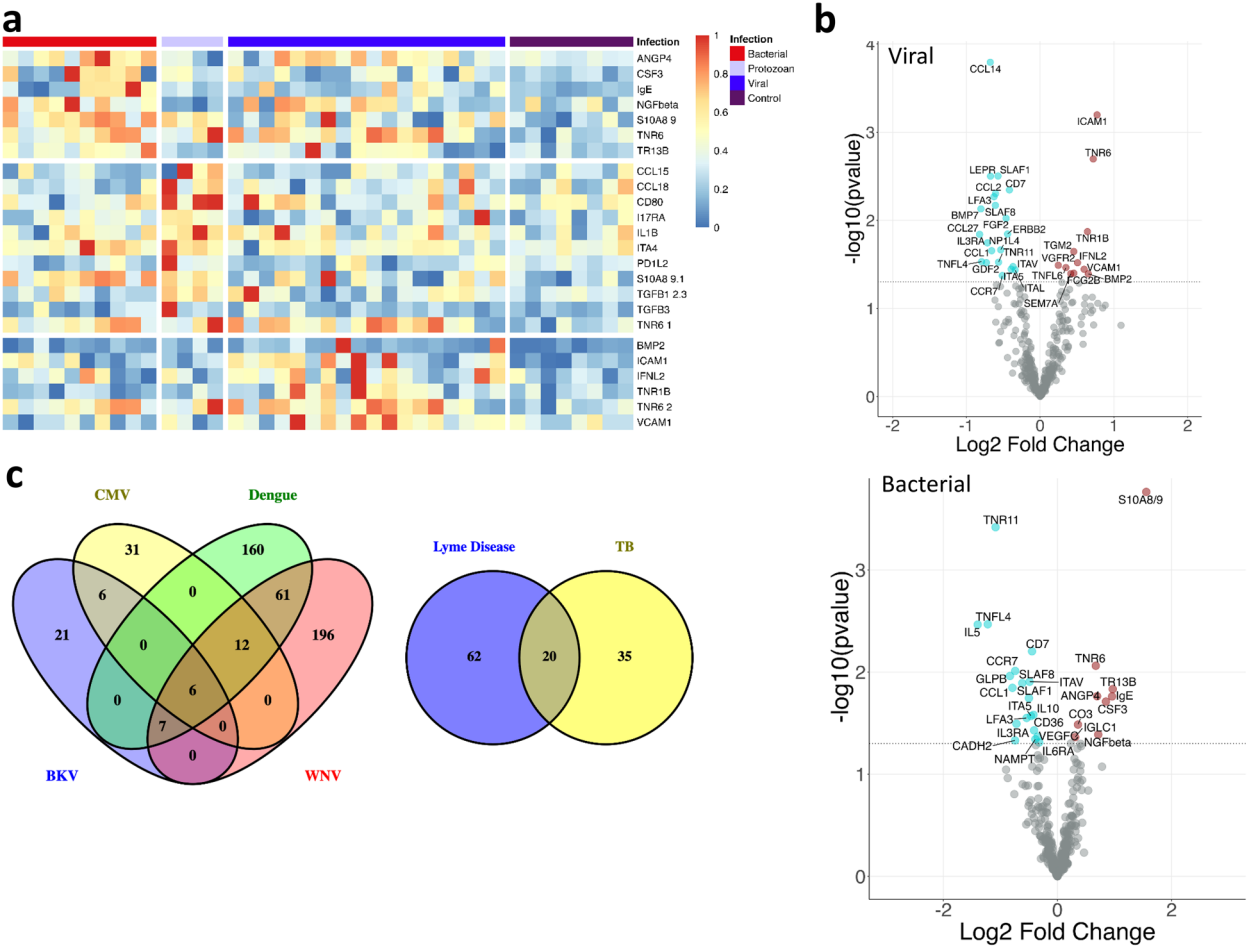
Analysis to identify significantly increased and decreased proteins and most enriched pathways in the acute infection phase compared to the healthy controls. **a** The heatmap shows the expression pattern of significant proteins in infection caused by each infectious agents (virus, bacteria, protozoan) with a fold change ≥ 1.4 compared to the healthy controls. **b** For each category, we identified several uniquely expressed proteins, with which we performed gene set enrichment analysis and identified the significantly affected biological processes at the time of infection.

Distinct and Common Protein Signatures at the Acute Infection Stage Compared to Healthy Controls Were Identified. We identified blood proteins whose expression changed during the acute infection phase when compared to their levels in the blood of healthy normal subjects. Three hundred forty-five blood proteins were analyzed in a multiplexed format with a high intra and intersample reproducibility and < 10% CV. The sensitivity of the assay ranges from 240 aM to 4 fM with a dynamic range of about 5 orders of magnitude. Tumor necrosis factor receptor 6 (TNR6) and C–C motif chemokine ligand 1 (CCL1) were increased in all bacterial, viral, and protozoan infections (Table 2) (full list in Table 2 supplementary). Proteins that were highly elevated in infections compared to controls include intercellular adhesion molecule 1 (ICAM1) [12–15], TNR6 [16], and vascular cell adhesion molecule 1 (VCAM1) [17] in viral infections; nerve growth factor beta (NGFβ) [18], TNR6 [19], and S100 calcium-binding protein A8/9 (S100A8/9) [20–22] in bacteria; CD80, integrin subunit alpha 4 (ITA4) [23], programmed cell death 1 ligand 2 (PD1L2) [24], S100A8/9 [25], transforming growth factor-beta (TGFB1) [26], and TNR6 [27] in protozoan infection. The top 10 proteins specifically increased in each infection type are listed in Fig. 2a. Twenty-one proteins’ expression was significantly changed (6 increased and 15 decreased) in viral infection. Expression of eighteen proteins was significantly changed (8 increased and 10 decreased) in bacterial infection. Twenty-six proteins’ expression was significantly altered (11 increased and 15 decreased) in protozoan infection (Table 2) (full list in Table 2 supplementary). With the cutoff threshold of ≤ 0.05 *p* value and 1.41-fold change, ICAM1 and CCL27 were the most significantly increased and decreased proteins, respectively, in the viral infection category (Fig. 2b). S100A8/9 and IL5 were the most significantly increased and decreased proteins, respectively, in the bacterial infection category (Fig. 2c). S100A8/9 and TNR16 were the most significantly increased and decreased proteins, respectively, in the protozoan infection category (Fig. 2d).

**Table 2:** List of Top 5 Increased and Decreased Blood Proteins Observed in Each Vector at Acute Infection

Protein	log2FC	*p* value	Disease
ICAM1	0.77	0.00	Viral
TNR6	0.72	0.00	Viral
BMP2	0.65	0.04	Viral
TNR1B	0.64	0.01	Viral
VCAM1	0.60	0.04	Viral
IL3RA	−0.71	0.02	Viral
GDF2	−0.73	0.03	Viral
TNFL4	−0.79	0.03	Viral
BMP7	−0.80	0.01	Viral
CCL27	−0.82	0.01	Viral
S100A8/9	1.56	0.00	Bacterial
TR13B	0.97	0.01	Bacterial
IgE	0.96	0.02	Bacterial
CSF3	0.85	0.02	Bacterial
NGFbeta	0.71	0.04	Bacterial
CCL1	−0.79	0.01	Bacterial
GLPB	−0.83	0.01	Bacterial
TNR11	−1.08	0.00	Bacterial
TNFL4	−1.22	0.00	Bacterial
IL5	−1.40	0.00	Bacterial
S10A8/9	1.34	0.01	Protozoan
TGFB3	0.98	0.01	Protozoan
CCL18	0.88	0.02	Protozoan
PD1L2	0.87	0.05	Protozoan
I17RA	0.85	0.03	Protozoan
PGH1	−1.03	0.01	Protozoan
CSF2	−1.10	0.02	Protozoan
GLPB	−1.17	0.01	Protozoan
IL3RB	−1.17	0.00	Protozoan
TNR16	−1.90	0.01	Protozoan

We performed gene set enrichment analysis on the detected proteins in each sample and identified biological processes that are significantly affected at the time of infection with the different pathogen groups. In bacteria-mediated infections, the most noteworthy biological processes were leukocyte migration (FDR = 1.7*E* − 12) and immune response (FDR = 6.45*E* − 12) (Table 3 supplementary). In viral infections, the most noteworthy biological processes were the cell surface signaling pathway (FDR = 12.58*E* − 13) and the response to external stimuli (FDR = 6.08*E* − 11) (Table 3 supplementary). In the protozoan category, the most noteworthy biological processes were the cell surface signaling pathway (FDR = 1.7*E* − 16) and the cytokine-mediated signaling pathway (FDR = 3.35*E* − 16) (Table 3 supplementary). Apart from these prominent pathways associated with each infection, there are 21 common pathways among all the infections (Table 3 supplementary).

**Table 3:** Significant Proteins (Top 5 Increased and Decreased) Observed in Each Vector at Convalescence Compared to Healthy Normal Control

Protein	logFC	*p* value	Disease
IgE	0.86	0.02	Bacterial
ANGP4	0.85	0.02	Bacterial
ADIPO	0.56	0.03	Bacterial
CD9	0.52	0.05	Bacterial
CCL27	−1.05	0.01	Bacterial
TNFL4	−1.09	0.01	Bacterial
IL5	−1.28	0.00	Bacterial
CD276	−1.44	0.04	Bacterial
CD45RB	−1.59	0.00	Bacterial
CCL18	0.90	0.03	Protozoan
IL17B	0.69	0.02	Protozoan
ITA4	0.68	0.01	Protozoan
CD80	0.51	0.02	Protozoan
IL3RB	−1.25	0.00	Protozoan
CSF2	−1.30	0.00	Protozoan
ONCM	−1.40	0.01	Protozoan
BMP5	−1.44	0.02	Protozoan
TNR16	−2.16	0.00	Protozoan
GLPA	0.97	0.04	Viral
ITAE	0.94	0.03	Viral
TNR6	0.77	0.01	Viral
NTF4	0.72	0.05	Viral
VEGF165, VEGF121	0.61	0.03	Viral
PDCD1	0.61	0.04	Viral
CCL14	−0.69	0.00	Viral
IL3RA	−0.70	0.01	Viral
CCL1	−0.71	0.01	Viral
IL17C	−0.73	0.04	Viral
IL5	−0.81	0.02	Viral

**Fig. 3:**
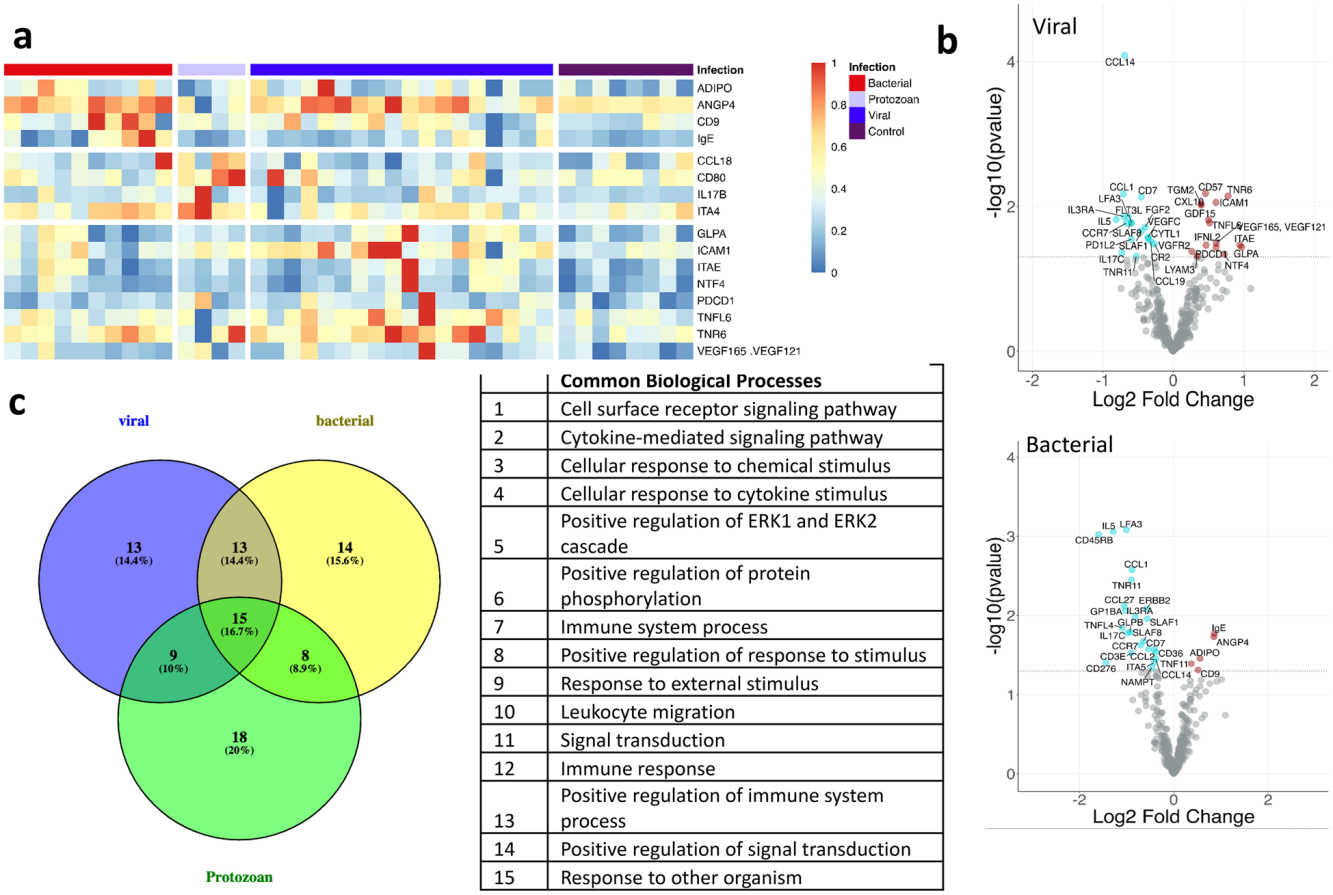
Analysis to identify differentially expressed proteins and most enriched pathways in the convalescent state or recovery phase of infection compared to the healthy controls. **a** The heatmap shows the expression pattern of top four significantly changed proteins in the convalescent or recovery phase of the infection per infectious agents (virus, bacteria, protozoan) with a fold change ≥ 0.5 compared to the healthy controls. **b** For each infectious agent, we identified several uniquely expressed proteins, with which we performed gene set enrichment analysis and identified the significantly affected biological processes at the time of recovery.

Protein Analysis Identifies Distinct and Common Protein Signatures in Profiled Samples at Convalescent Stage Compared to the Healthy Controls. Next, we compared the expression pattern of significantly increased or decreased proteins at the convalescent stage with a fold change of equal to or higher than 1.41-fold in comparison to healthy controls (Fig. 3a, Table 3) (Table 4 supplementary). C–C motif chemokine ligand 1 (CCL1) and C–C Motif Chemokine Receptor 7 (CCR7) were increased in all bacterial, viral, and protozoan infections. Proteins that were highly elevated in the recovery or convalescent stage compared to healthy controls included CD57, TNR6, and tumor necrosis factor ligand (TNFL6) in viral infection. CCL14 and CD7 were among some of the most decreased proteins in viral infection (Fig. 3b). Angiopoietin 4 (ANGP4), CD9, and immunoglobulin E (IgE) were increased and LFA3, IL5, and CD45RB were among the most decreased proteins in bacterial infection (Fig. 3b). ITA4, CD80, C–C motif chemokine ligand 18 (CCL18), and IL17B were among the increased and PGH1, IL3RB, and IL25 were among the decreased proteins in protozoan infection (Fig. 2 supplementary).

To assess enriched biological processes at the convalescent state of the infections, we performed gene set enrichment analysis. In bacteria-mediated infections, the most noteworthy biological processes were the immune system process (FDR = 4.49*E* − 11) and the positive regulation of protein phosphorylation (FDR = 3.52*E* − 10) (Table 5 supplementary). In viral and protozoan infections, the most noteworthy biological processes were the cell surface signaling pathway (FDR = 3.58*E* − 8) and the cytokine-mediated signaling pathway (FDR < 1.18*E* − 8) (Table 5 supplementary). There were 15 common pathways across all the infections (Table 5 supplementary).

**Fig. 4:**
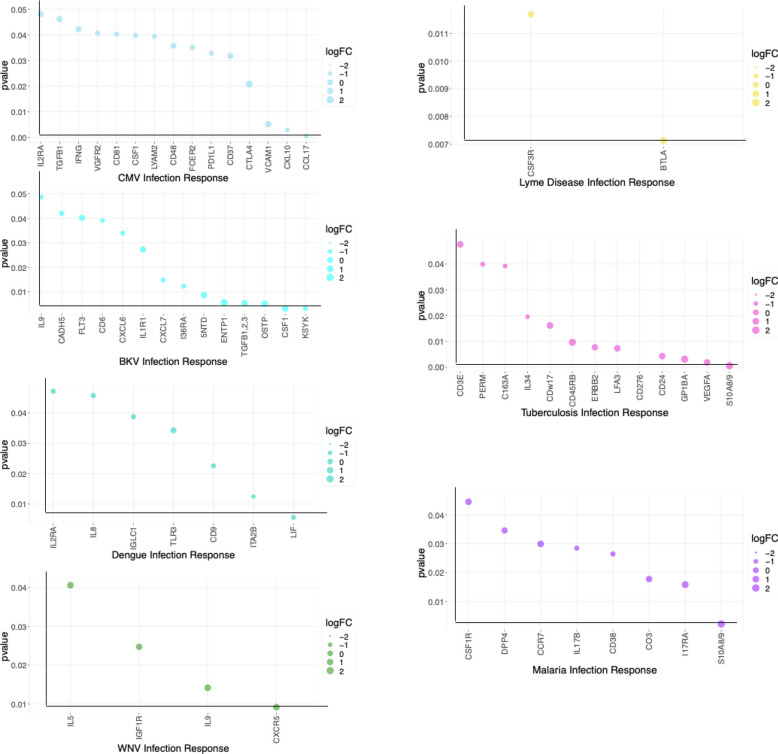
Significantly increased and decreased blood proteins in each infection type were identified by comparing the acute infection phase to the convalescent phase. The bubble plot shows their names and the degree of their perturbation during acute infection time points.

**Fig. 5:**
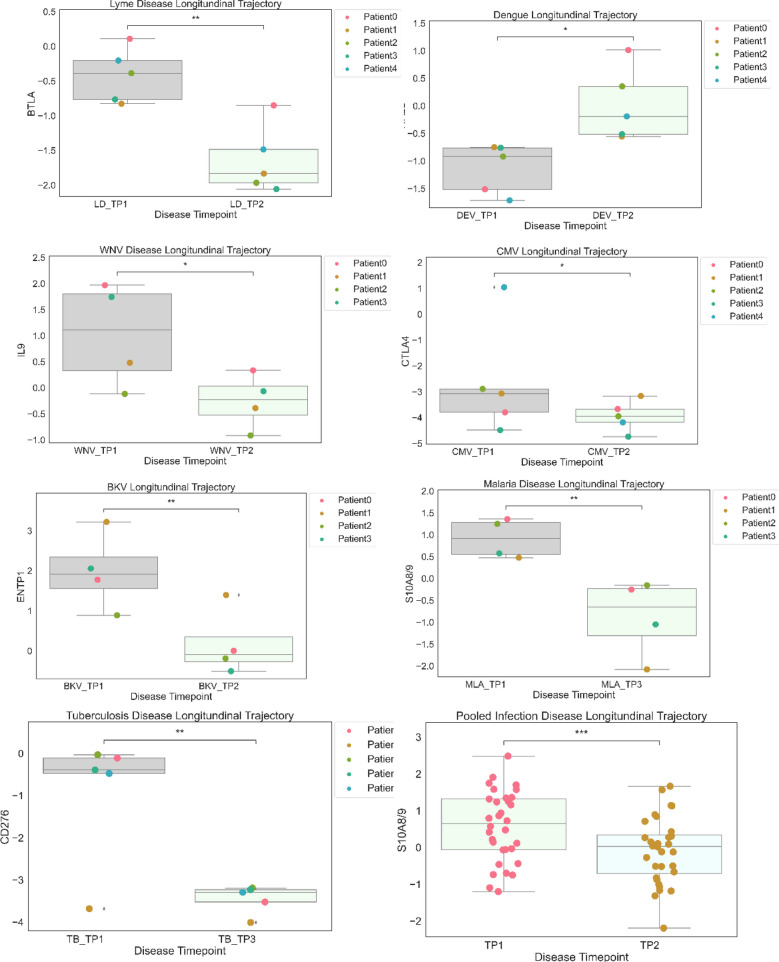
The boxplots display the most differentially changed proteins across the time points of each infection. Non-significant (ns): *p* < = 1.00*e* + 00; *1.00*e* − 02 < *p* < = 5.00*e* − 02; **1.00*e* − 03 < *p* < = 1.00*e* − 02; ***1.00*e* − 04 < *p* < = 1.00*e* − 03; ****p < = 1.00*e* − 04.

Distinct and Common Protein Signatures Comparing Acute and Convalescent Stages of All the 7 Infections. We identified differentially expressed blood proteins in each infection type. There were multiple proteins which were either elevated or depleted at the time of acute infection (Table 6 supplementary). A bubble plot in Fig. 4 shows their names and the degree of their perturbation during acute infection time points. The heatmap in Supplemental Fig. 4a shows the top elevated plasma proteins in the acute phase compared to the convalescent stage for each infection. We have identified the proteins with the highest difference in expression between acute infection and a time point at convalescent phase for each disease and showed the perturbation as longitudinal plots in Fig. 5.

We first looked at the proteins identified as being specific to viral infection. IL-9, produced by T cells, has a trend to be upregulated in the acute phase of WNV infection, suggesting that IL-9 may have a role in host defense or viral pathogenicity. In our study, all the CMV-positive patients underwent a kidney transplant and had higher expression of immune checkpoint protein CTLA4 (cytotoxic T lymphocyte-associated protein 4) in the acute infection stage. Expression of ectonucleoside triphosphate diphosphohydrolase 1 (ENTP1) or CD39 protein which catalyzes the hydrolysis of ATP is significantly enhanced in the acute phase of BKV infection. Increased expression of CTLA4 and ENTP1 could be a side-effect of the immunosuppressive drugs that kidney transplant patients are currently prescribed. Plasma BKV positivity is highly associated with co-infection CMV, suggesting possible risk factors for these infections [28]. Chemokine C–C motif chemokine ligand 27 (CCL27) is downregulated in the acute stage of viral infection. Its association with viral infection at the acute or convalescent stage has never been studied in the pathogenesis of viral infections. In DENV, integrin subunit a, integrin subunit alpha 2b (ITA2b or CD41) is crucial for blood coagulation. We observed that ITA2b is significantly downregulated in the acute phase of DENV infection.

Several proteins demonstrated their association with bacterial infection. In LD, immune checkpoint protein B and T lymphocyte attenuator (BTLA) level significantly increased in the acute phase compared to the convalescent period. CD276 level is increased in acute phase of TB compared to convalescent state. Healthy controls have elevated levels of IL-5 compared to individuals with acute bacterial infections. Furthermore, patients with acute bacterial infection continue to have low IL-5 concentrations in the convalescent stage (Fig. 4c supplementary).

Our analysis identified TNR16/CD271, receptor expressed by mesenchymal stem cells to be downregulated in the acute and convalescent stages of malaria. However, to date, TNR16/CD271 has not been reported as being associated with malaria.

We report here higher levels of S100A8/A9 at the time of acute microbial infection and its decline at the convalescent stage supporting its antimicrobial and chemotactic property. Since S100A8/A9 is involved in inflammatory processes induced either by pathogens or in chronic inflammation such as tendinopathy [29], it is an indicator of inflammation rather than one diagnostic for a specific pathogen. A change in its concentration in serum from acute to convalescent phase, however, could serve as a potential diagnostic marker for microbial infection. These calcium-binding proteins are highly expressed in neutrophils and cause neutrophil chemotaxis, which are involved in the immune defense [30]. S100A8 is a marker of neutrophil-mediated inflammation, and there are studies suggesting it as a useful biomarker to distinguish bacterial from viral respiratory infections [31].

Pathway analysis demonstrated that regulating the immune system process was the most significantly enriched biological process (*p* = 3.1*E* − 22). Biological processes enriched in individual infections and in bacterial and viral infections are listed in Table 7 supplementary. There are 21 biological processes commonly enriched in between bacterial and viral infections (Table 7 supplementary). Three selected proteins and their relative abundance in viral, bacterial, and protozoan infections at the time of acute infection when compared to the convalescent state are presented in Fig. 4c supplementary.

## DISCUSSION

We have performed a comprehensive analysis of circulating proteins in human cohorts infected with phylogenetically distinct pathogens, with the goal of identifying common and unique proteins, mediators, and pathways that contribute to the pathophysiology of infection. The high-throughput proteomic profiling used in this study allowed for sample analytic multiplexity without sacrificing sensitivity and data reproducibility. In this unique pilot study design, we used paired samples from seven different human infections caused by multiple viruses, bacteria, and protozoa to assess proteins and infer pathways activated during active infection and recovery and identified common and unique proteomic profiles of responses to a range of different pathogens.

Human viruses are known to develop suitable strategies for modifying apoptosis in virus-infected cells and in virus-primed T cells. Apoptosis-mediated physiological depletion of T lymphocytes due to viral infection silences the immune response [32]. IL-2, IFN-g, or TNF-a is known to induce the co-expression of TNR6 and ICAM1 on activated B cells. This might suggest a role of ICAM1 and ICAM1-mediated signals in the induction of B cell apoptosis [33, 34]. VCAM1 is another cell-adhesion molecule, known to play a role in regulating T cell-mediated inflammation and pathology, found to be up in the acute phase of viral infection [17]. Thus, our findings underscore the potential role of VCAM-1 in regulating the immune response and inflammatory reactions against viral infections.

Both IgE and ANGP4 are up in the acute and recovery phases of bacterial infection. Most LD patients produce specific IgE antibodies [35], and its concentration is usually higher in patients with TB [36]. ANGP4 promotes angiogenesis, which is crucial in tick-borne encephalitis [37, 38]. Increased expression of CSF3 is uniquely associated with acute bacterial infection, which can be explained as CSF3 is known to be a biomarker for the pathogenesis of the cytokine storm in active TB [39]. Remission of tuberculosis infection could be ascertained by the expression of CD9, an exosome marker, one of the highly expressed proteins in the recovery phase of bacterial infection cohort [40].

Previous studies have reported infection-specific proteins that distinguish bacterial infections from viral [41]. TNR6, a TNF-receptor superfamily expressed on the cell surface of activated human T and B lymphocytes [42], was a common protein upregulated during all acute infections and continues to be up during the recovery phase in viral infection (Figs. 2 and 3). TNR6-mediated apoptosis may have a role in the induction of peripheral tolerance, in the antigen-stimulated suicide of mature T cells, or both [43]. More recently, TNR6 has been reported to be involved in inflammation or cell migration [44]. It was interesting to note that TNR6 continued to be upregulated in the recovery phase of viral infection along with its death ligand TNFL6 and ICAM1.

In malarial infection, levels of CD80 and CCL18 are elevated in both the acute and recovery phases. CD80 is expressed on memory B cells essential for maintaining long-term humoral immunity to infectious organisms, including Plasmodium [45, 46]. Host responses against Plasmodium involve humoral and cell-mediated immunity. Higher expression of S100A8/9 protein in the acute phase of the Plasmodium-infected patients was also accompanied by increased abundance of CCL18 in the convalescent stage, a cytokine produced by myeloid cells that helps in T cell-mediated immunity (Figs. 2 and 3) [47] and tissue repair processes at epithelial surfaces. These myeloid cells also manufacture IL17B that facilitates the communication between leukocytes and epithelial cells, enhancing innate defense mechanisms and tissue repair processes at epithelial surfaces [48, 49]. Knowledge of the biological functions of this cytokine is limited. In our study, IL17B is upregulated during the recovery phase of malaria and, therefore, might possess a defensive role. In malaria infection, MSCs counteract parasite-mediated negative regulation of T cell functions [50].

The Ca + 2 and Zn + 2 binding heterodimeric protein S100A8/9 was a common protein family upregulated during active infections in bacteria and malaria, with the greatest decline during convalescence. Under physiological conditions, these proteins are stored in neutrophils, monocytes, and macrophages, secreted upon tissue/cell damage or cellular stress or activation of phagocytes, serving as a danger signal [51, 52]. Here in inflammatory conditions, we observe that irrespective of the pathogen type, a surge in these proteins is a hallmark of acute infection. S100A8/A9 effectively inhibits the growth of bacteria *Borrelia* at infectious sites during the initial phase of LD [20], allowing time for the recruitment of phagocytes. Subsequently, S100A9 enhances the phagocytic activity of infiltrating leukocytes, accelerating the clearance of pathogens [21]. Also, targeting S100A8/A9 reduces organ injury by decreasing tissue damage in the lung during TB [22]. S100A8/A9 is found to be elevated in the serum of malaria patients, which may contribute to pathogen immune escape or tolerance [25]. Therefore, we conclude that S100A8/A9 is induced in two infectious diseases studied here and may play a modulatory role in acute infection caused by bacteria and protozoa. Further studies would be needed to evaluate this marker in the context of infection recovery vs resistance, which is beyond the scope of this study.

In the acute phase of all the infections compared to the healthy control, we have identified 21 common upregulated signaling pathways (Fig. 2 supplementary, Table 3 supplementary)—cell surface receptor signaling, response to external stimulus, and cytokine-mediated signaling pathway are few to mention. Understanding of such interactions between the pathogen and host is critical for guiding novel therapies and understanding the factors that lead to the development of active disease. Cell surface receptor signaling is a crucial step where once the bacteria adhere to the surface they exert greater resistance toward clearance by immune factors and establish pathogenesis [53]. In TB, *M. tuberculosis* binds to cell surface receptors and shifts the balance from the host‐protective apoptotic cell death program toward a lytic form of host cell death to orchestrate the infection process to facilitate its growth, dissemination, and entry into latency [54]. In case of LD, *B. burgdorferi* activates several intracellular pathogen recognition receptors including cell surface TLRs, which collectively contribute to inflammatory signaling [55, 56]. In malaria, Plasmodium’s entry into the host erythrocytes to establish its pathogenesis requires multiple molecular interactions between the surface proteins of merozoites and cell surface receptors on the host including TLRs and glycosylphosphatidylinositol-mediated signaling [57, 58]. Viruses overcome intrinsic cellular barriers by inducing distinct classes of receptor-mediated endocytosis coupled with receptor-mediated signaling [59]. Virus–receptor interactions play a key regulatory role in viral host range and viral pathogenesis [60]. Integrins are known to function as the cellular receptor for WNV [60], gB and gH/gL are essential for CMV entry [61], sulfated glycosaminoglycans (GAGs), lectins, glycosphingolipid (GSL), proteins with chaperone activity, laminin-binding proteins, are known to be crucial for DENV [62].

Downregulation of IL-5, CCL27, and TNR-16 proteins is unique to bacterial, viral, and malarial infections, respectively, and could provide pathogen-specific monitoring biomarkers for early and recovering infections. TNR-16/CD271 is a surface marker of MSCs and is downregulated in the acute and convalescent phases of malaria (Fig. 3 supplementary). This suggests reduced capacity to combat immune modulation by the pathogen.

In the acute phase of malaria, P-glycoprotein homolog 1 (PGH1) is downregulated, a protein of Plasmodium falciparum, which has been linked to chloroquine, mefloquine, and halofantrine resistance [63]. This may indicate that our cohort is sensitive to antimalarials. In the convalescent phase of malaria, BMP5 a ligand of the TGF-beta superfamily that can lead to the recruitment and activation of SMAD family transcription factors [64] is downregulated. More research is needed to determine whether TGF-beta levels could be a candidate marker for malarial infection or disease severity.

In the acute phase of bacterial infection, TNR11, a TNF-receptor superfamily, is downregulated. They are essential regulators of the interaction between T cells and dendritic cells. Downregulation of TNR11 may impair the differentiation of naive CD4 + T cells toward Th2 cells contributing to the chronic tissue-destructive T cell activity [65]. In the convalescent phase of bacterial infection, CD45RB, CD276, and CCL27 are downregulated. CD45 is a receptor tyrosine phosphatase essential for TCR signaling [66], CD276 belongs to the immunoglobulin superfamily that causes T cell activation and IFN-γ production [67], and CCL27 is a cytokine pivotal role in T cell-mediated inflammation [68]. This indicates that during bacterial infection and recovery, T cell response and activation are low, which may indicate that it has a minor role in defending against bacteria. It was interesting to note that CCL27 and BMP7 are down in the acute phase of viral infection as this may result in amplified virus infection. CCL27-knockout mice have overreactive skin inflammatory responses in a model of psoriasis [69]. BMP7 is a potent antagonist of TGF-β1 and an antifibrotic [70]. In the convalescent phase, IL17C and CCL1 got our attention as both were significantly downregulated. IL17C is a proinflammatory cytokine that reinforces innate immune barriers and stimulates highly inflammatory TH17 cells involved in the pathogenesis of several diseases, including infectious [71]. CCL1 is involved in inflammatory processes through leukocyte recruitment and could play a crucial role in angiogenesis and other viral and tumoral processes [72]. BTLA is the most significantly elevated protein in acute Lyme disease infections. Its expression is high on terminally differentiated B and T cells, where it is known to repress the activation of signal transduction. Moreover, BTLA expression may indicate terminally exhausted lymphocytes. BTLA shares similarities with other immune checkpoints such as PD-1 and CTLA-4 which are the targets of the currently used immunotherapies [73].

Several similar studies have reported protein and gene transcripts associated with the pathogens included in our study [5, 74–76]. These studies include basic transcriptomics studies to LC–MS-based platforms to modified aptamer-binding technology (“SOMAmers”). For example, Penn-Nicholson et al. identified signatures for TB including 5-protein signature that included C9, IGFBP-2, CD79A, MXRA-7, and NrCAM. These proteins were elevated in TB progressors compared to non-progressors [74]. The SomaScan Assay included ~ 3000 proteins and the platform in this study included ~ 350 proteins. Of them, only 40 proteins were common in the two platforms. Perhaps because of the study design and the assay platforms the TB-specific proteins identified in Penn-Nicholson et al. and ours do not overlap. The markers proteins. Liu et al. performed [75] reported an 8-gene marker panel to improve clinical prediction of severe dengue progression that included LTF, UQCRQ, CKAP4 as increased and ARNTL, PDGFRB, TGFBR3, RASSF5, GDPD5 as decreased [75]. These transcripts also were not significant in our study, partly, it was a gene expression study, and we profiled proteins, and we did not seek progression markers in our study. Recently, we have published protein signatures on LD and WNV cohorts for their specific markers by using two LC–MS-based proteomic protocols which also identified a different unique set of proteins [5]. The non-overlap of markers is due to the unique antibody-based platform specifically designed to capture cell surface marker and cytokine profiling. The unique discoveries made in these studies could complement each other and provide an overall picture of the pathobiology for further validation.

We acknowledge a few limitations in this study. We used serum and plasma to profile host proteins in different diseases. Even though previous proteomic studies have suggested essentially the same proteomic profiles for serum and plasma, there are differences in clotting factors and fibrinogens, which may affect our ability to compare different cohorts and diseases [77]. Another limitation of our study is the small sample size. The study was part of an ancillary project where the objective was to assess the usefulness of “omic” interrogation of circulating proteins in the blood of different infectious agents. The samples were collected from studies associated with the NIH/NIAID HIPC consortium. As we have listed the studies and centers in the “Materials and Methods” section describing the study subjects and samples, this presents a unique opportunity to assess proteomic profiling of the biobanked blood samples. Since the data presented here was generated from a set of pathogens by design and due to the limited sample numbers, the specificity of the markers identified may not represent all viral, all bacterial, and all microbial infections.

In conclusion, we evaluated the common and unique proteomic profiles of responses to infection by a range of different pathogens. These observations and markers will help generate hypotheses that can be tested by designing studies with larger cohorts to validate the clinical significance of these markers.

## MATERIALS AND METHODS

Study Subjects and Samples. The study includes 72 blood (serum or plasma) samples collected from 32 subjects infected with seven different pathogens, obtained through collaboration with six leading academic centers in the US, that participated in the Human Immunology Project Consortium (HIPC) (Fig. 1a). The research has been carried out in accordance with the institutional review board approval, and all the participants provided written informed consent. We received BK virus nephropathy (BKV) samples (*n* = 4, male = 2, female = 2, mean age = 53.75 years) from kidney transplant recipients consented at UCLA through IRB#11–001387. Cytomegalovirus blood samples (*n* = 5, male = 2, female = 3, mean age = 38 years) were collected from kidney transplant recipients enrolled at UCSF through IRB approved study #14–13,573 and NIH 3U19AI1281913 study entitled “Mapping Immune Responses to CMV in Renal Transplant Recipients.” Study samples from patients with malaria (*n* = 4, male = 2, female = 2, mean age = 32.5 years) were obtained from Seattle Children’s Research Institute through IRB#20,162,556. The samples were collected as a part of the Immunization by Mosquito with Radiation Attenuated Sporozoites (IMRAS) trial (NCT01994525). The samples in the study were from the four infection control subjects in the IMRAS trial, i.e., received mosquito bites but no attenuated parasites (i.e., vaccine) during the vaccination phase, and then were challenged with wild-type parasites and became infected. The time points are acute infection (day 5/6 post-challenge infection) and convalescent (day 112 post-challenge infection and following drug treatment ~ day 11 to resolve the infection). Samples from the tuberculosis cohort (*n* = 5, male = 4, female = 1, mean age = 38.25 years) were obtained from La Jolla Institute for Immunology through IRB#VD-143. Dengue study participants (*n* = 5, male = 2, female = 3, mean age = 9.4 years) in Managua, Nicaragua, were consented through UC Berkeley IRB #2010–06-1649 and the Nicaraguan Ministry of Health IRB# NIC-MINSA/CNDR CIRE-01/10/06–13. The blood samples were collected as part of the Pediatric Hospital-based Dengue Study in Nicaragua, with multiple funding sources since 2005 (ongoing). Two recent funding sources are NIH grants P01 AI106695 (EH) and U19 AI118610 (EH/AS—HIPC). Samples from the West Nile virus cohort (*n* = 4, male = 2, female = 2, mean age = 55.75 years) studies were obtained through H-305333 and 0510000728 at Baylor College of Medicine and Yale School of Medicine. The Lyme disease cohort (*n* = 5, male = 2, female = 3, mean age = 61.6 years) was obtained through IRB# 1,112,009,475 at Yale School of Medicine. Both WNV and LD samples were collected as a part of HIPC grant entitled “Systems Immune Profiling of Divergent Responses to Infection” (NIH award AI 089992). Healthy control (*n* = 4, male = 2, female = 2, mean age = 43.75 years) samples were also included for comparison. All samples were stored at −20° C until use.

ScioCD Assay. In brief, 72 blood samples (Fig. 1a) were labeled at an adjusted protein concentration for 2 h with the fluorescent dye scioDye 2 (Sciomics, Neckargemünd, Germany). Sample labeling and incubation were performed as previously described in detail [78, 79]. In brief, we prepared a reference sample by pooling all samples included and labeled with a second dye (scioDye 1). After 2 h, the labeling reaction was stopped, and the buffer exchanged to phosphate-buffered saline (PBS). For improved assay robustness and differentiation power, each sample was competitively incubated together with a common reference sample on one microarray slide in a reference-based dual-color approach as described in detail before [80]. The samples were then assayed on scioCD antibody microarrays (Sciomics) targeting 351 different proteins by 517 antibodies, each in four replicates. Array surfaces were blocked with scioBlock (Sciomics) on a Hybridization Station 4800 PRO (Tecan, Grödig, Austria), and the samples were subsequently incubated competitively with the reference sample using a dual-color approach. After incubation for 3 h, the slides were thoroughly washed with 1 × PBSTT (phosphate-buffered saline containing Tween and Triton), rinsed with 0.1 × PBS as well as with water and subsequently dried with nitrogen.

Data Acquisition and Analysis. Slide scanning was conducted using a Powerscanner (Tecan, GmbH, Grödig, Austria) with constant instrument laser power and photomultiplier settings. Spot segmentation was performed with GenePix Pro 6.0 (Molecular Devices, Union City, USA). The median signal intensities of the spots were used. The background was not subtracted as an earlier one, and continuous assessments proved for antibody microarray analyses that background subtraction is not reducing but inducing artifacts. The acquired raw data were analyzed using the linear models for microarray data (limma) package of R-Bioconductor after uploading the median signal intensities. Isoforms in the dataset were merged and replaced with mean value resulting in 345 unique proteins (Table 1 supplementary). For normalization, a specialized invariant Lowess method was applied [80]. A linear model was fitted using limma, and differences between sample groups were calculated based on the fitted group means generated by the linear model and are presented as log-fold changes (logFC) calculated for the basis 2.

Data Analysis. Data was analyzed with the objective of identifying differences between (1) active infection state and healthy controls, (2) convalescent state and healthy controls, and (3) active infection state and convalescent state. Preprocessing and analysis were conducted by using Python (3.8.8) and R (4.0.4). Acquired raw data were analyzed using the linear models for the microarray data (LIMMA 3.46) package of R-Bioconductor after uploading the median signal intensities. For normalization, a specialized invariant Lowess method was applied. For analysis of the samples, a multi-factorial linear model was fitted with LIMMA resulting in a two-sided *t*-test or F-test based on moderated statistics. Multiple contrasts were defined between disease timepoints and disease classes to discern protein signatures. Differential expression results were represented by using the R packages ggplot2 (3.3.5) and pheatmap (1.0.12). Next to infection type, the sample type was accounted for using the information as an additional factor in the linear model. All presented *p* values were adjusted for multiple testing by controlling the false discovery rate according to Benjamini and Hochberg. Proteins were defined as differential for logFC > 0.5 (1.4 fold change) and an adjusted *p* value < 0.05. Moreover, descriptive statistics were captured by using the Python packages matplotlib (3.3.4), numpy (1.19.5), pandas (1.2.0), scipy (1.7.2), and seaborn (0.11.1). Comparisons for disease signature were defined by observing differences between the initial acute disease timepoint and the final recovery timepoint. The healthy control cohort (*n* = 4) was normalized using the R package sva (3.38.0) to standardize observed variation with Combat. Differences in protein abundance between samples or sample groups are presented as log-fold changes (logFC) calculated for the base 2. Gene set enrichment was conducted on the statistically significant proteins (*p* value <= 0.05) through the Python package gseapy (0.10.4) observed from the comparison of acute infection timepoint with the healthy control as well as the disease in comparison to the healthy control. Combined score is described as *c* = log(*p*) * *z*, where *c* is the combined score, *p* = is the Fisher exact test *p* value, and *z* is the *z*-score for deviation from expected rank (81). Gseapy utilized the human catalog from KEGG 2021, GO Biological Process 2021, as well as GO Molecular Function 2021. The study schematic is presented in Fig. 1b.

### Supplementary Information

Below is the link to the electronic supplementary material.

Supplementary file1 (DOCX 32 KB)

Supplementary file2 (XLSX 41 KB)

Supplementary file3 (XLSX 13 KB)

Supplementary file4 (XLSX 77 KB)

Supplementary file5 (XLSX 61 KB)

## Data Availability

Proteomics data generated from this study has been deposited in IMMPORT database with accession number SDY2008, under workspace ID 6886 “HIPC Multicenter Acute Infection IOF.”
