# Supplementary material for: Proteome Analysis for Inflammation Related to Acute and Convalescent Infection
Source: Inflammation. Author manuscript; Available in PMC 2024 Feb 5. (PMC10799112; doi:10.1007/s10753-023-01913-3)
Supplement: Supplementary File1 [file NIHMS1956755-supplement-Supplementary_File1.docx]

**Supplemental Data:**

**Supplemental Table 1.** List of 345 proteins assays by the ScioCD Assay platform.

(This is provided as a separate Excel spreadsheet)

**Supplemental Table 2:** List of increased and decreased blood proteins observed in each vector at acute infection

| **Protein** | **logFC** | **pvalue** | **Disease** |
| --- | --- | --- | --- |
| S100A8/9 | 1.556034038 | 0.000172219 | Bacterial |
| TR13B | 0.968643155 | 0.014666334 | Bacterial |
| IgE | 0.956945226 | 0.017303037 | Bacterial |
| CSF3 | 0.849023813 | 0.019405829 | Bacterial |
| NGFbeta | 0.714309161 | 0.04088211 | Bacterial |
| ANGP4 | 0.694693236 | 0.017223097 | Bacterial |
| TNR6 | 0.671352277 | 0.00866789 | Bacterial |
| SLAF1 | -0.500428075 | 0.017969476 | Bacterial |
| LFA3 | -0.540717054 | 0.028117336 | Bacterial |
| SLAF8 | -0.617980885 | 0.012814711 | Bacterial |
| IL3RA | -0.718285341 | 0.032047616 | Bacterial |
| CCR7 | -0.741290456 | 0.009736502 | Bacterial |
| CADH2 | -0.743540642 | 0.046635539 | Bacterial |
| CCL1 | -0.78989475 | 0.01425057 | Bacterial |
| GLPB | -0.833555014 | 0.010917374 | Bacterial |
| TNR11 | -1.084927045 | 0.000381586 | Bacterial |
| TNFL4 | -1.22189711 | 0.003401135 | Bacterial |
| IL5 | -1.398406055 | 0.003423681 | Bacterial |
| S10A8/9 | 1.340940854 | 0.008550334 | Protozoan |
| TGFB3 | 0.976363542 | 0.006213694 | Protozoan |
| CCL18 | 0.883913047 | 0.018226488 | Protozoan |
| PD1L2 | 0.866986945 | 0.047081493 | Protozoan |
| I17RA | 0.853827113 | 0.032067255 | Protozoan |
| TNR6 | 0.780414324 | 0.017208752 | Protozoan |
| CCL15 | 0.732185964 | 0.038901148 | Protozoan |
| IL1B | 0.714162827 | 0.013875313 | Protozoan |
| ITA4 | 0.635533491 | 0.015485513 | Protozoan |
| TGFB1,2,3 | 0.570719201 | 0.030829255 | Protozoan |
| CD80 | 0.562392683 | 0.006506084 | Protozoan |
| KSYK | -0.514181698 | 0.029897117 | Protozoan |
| HLAG | -0.525386918 | 0.030092262 | Protozoan |
| FGF2 | -0.568184501 | 0.025205604 | Protozoan |
| DPB1 | -0.624612391 | 0.009470011 | Protozoan |
| CD15 | -0.764681968 | 0.034275503 | Protozoan |
| LEPR | -0.853423182 | 0.008581975 | Protozoan |
| CCL1 | -0.856282571 | 0.037486859 | Protozoan |
| IL2 | -0.889872806 | 0.007246227 | Protozoan |
| IL25 | -0.92650265 | 0.005358925 | Protozoan |
| CD28 | -0.993081378 | 0.024867775 | Protozoan |
| PGH1 | -1.025532988 | 0.005170863 | Protozoan |
| CSF2 | -1.097170504 | 0.016593113 | Protozoan |
| GLPB | -1.166602101 | 0.006127522 | Protozoan |
| IL3RB | -1.168202299 | 0.002805868 | Protozoan |
| TNR16 | -1.895698587 | 0.008315 | Protozoan |
| ICAM1 | 0.771983458 | 0.000635892 | Viral |
| TNR6 | 0.719971667 | 0.002011845 | Viral |
| BMP2 | 0.652808263 | 0.040417818 | Viral |
| TNR1B | 0.641368832 | 0.013432155 | Viral |
| VCAM1 | 0.596375145 | 0.035885824 | Viral |
| IFNL2 | 0.506839616 | 0.030239069 | Viral |
| CCR7 | -0.512949179 | 0.042342488 | Viral |
| NP1L4 | -0.534374659 | 0.021610066 | Viral |
| TNR11 | -0.564239293 | 0.029859526 | Viral |
| SLAF1 | -0.570995784 | 0.003143623 | Viral |
| CCL2 | -0.604824313 | 0.004965891 | Viral |
| SLAF8 | -0.606932715 | 0.006758135 | Viral |
| LFA3 | -0.625614805 | 0.005391842 | Viral |
| CCL1 | -0.657420994 | 0.022110384 | Viral |
| LEPR | -0.673094803 | 0.003153604 | Viral |
| CCL14 | -0.67661515 | 0.000161049 | Viral |
| IL3RA | -0.714501112 | 0.018016781 | Viral |
| GDF2 | -0.72913409 | 0.030177865 | Viral |
| TNFL4 | -0.793874944 | 0.029416148 | Viral |
| BMP7 | -0.803457399 | 0.007406391 | Viral |
| CCL27 | -0.820844364 | 0.014415224 | Viral |

**Supplemental Table 3.** Detail description of the associated pathways

(This table is provided as a separate Excel spreadsheet)

**Supplemental Table 4:** Significant proteins (Increased and decreased) observed in each vector at convalescence compared to healthy control

| **Protein** | **logFC** | **pvalue** | **Disease** |
| --- | --- | --- | --- |
| IgE | 0.858346376 | 0.016982585 | Bacterial |
| ANGP4 | 0.85024019 | 0.018641268 | Bacterial |
| ADIPO | 0.55788274 | 0.034969317 | Bacterial |
| CD9 | 0.515456415 | 0.048706779 | Bacterial |
| CCL2 | -0.53740544 | 0.02657697 | Bacterial |
| SLAF1 | -0.561266154 | 0.010993575 | Bacterial |
| ERBB2 | -0.57664124 | 0.008231362 | Bacterial |
| SLAF8 | -0.643671986 | 0.021162247 | Bacterial |
| CCR7 | -0.701940025 | 0.023751061 | Bacterial |
| IL3RA | -0.82024115 | 0.010298906 | Bacterial |
| CCL1 | -0.885555916 | 0.002656324 | Bacterial |
| TNR11 | -0.898301971 | 0.003532133 | Bacterial |
| CD3E | -0.900152111 | 0.029701846 | Bacterial |
| GLPB | -0.92547931 | 0.016236554 | Bacterial |
| IL17C | -0.973530999 | 0.016710282 | Bacterial |
| LFA3 | -1.001798768 | 0.000826073 | Bacterial |
| GP1BA | -1.028337361 | 0.008678328 | Bacterial |
| CCL27 | -1.051637947 | 0.007506571 | Bacterial |
| TNFL4 | -1.090064778 | 0.014379973 | Bacterial |
| IL5 | -1.281948718 | 0.00087127 | Bacterial |
| CD276 | -1.441898667 | 0.039469054 | Bacterial |
| CD45RB | -1.591915407 | 0.000940171 | Bacterial |
| CCL18 | 0.904020817 | 0.031928993 | Protozoan |
| IL17B | 0.692410455 | 0.024199915 | Protozoan |
| ITA4 | 0.684977227 | 0.006133842 | Protozoan |
| CD80 | 0.514360329 | 0.015887806 | Protozoan |
| CD37 | -0.649160506 | 0.037433088 | Protozoan |
| CO3 | -0.65101844 | 0.034086779 | Protozoan |
| CD20 | -0.652553605 | 0.019627592 | Protozoan |
| CCL20 | -0.678089283 | 0.020015047 | Protozoan |
| FLT3L | -0.761862786 | 0.031777623 | Protozoan |
| HLAG | -0.797021472 | 0.008742813 | Protozoan |
| CCR7 | -0.803545459 | 0.04354868 | Protozoan |
| CD15 | -0.865753594 | 0.017688286 | Protozoan |
| IL25 | -1.020506943 | 0.001467013 | Protozoan |
| CCL1 | -1.035320801 | 0.006002467 | Protozoan |
| ANGP4 | -1.062700148 | 0.022460795 | Protozoan |
| CSF1R | -1.094869574 | 0.02168411 | Protozoan |
| PGH1 | -1.145636461 | 0.000332649 | Protozoan |
| GLPB | -1.153095465 | 0.02005982 | Protozoan |
| IL3RB | -1.247589172 | 0.000515916 | Protozoan |
| CSF2 | -1.295311216 | 0.001828499 | Protozoan |
| ONCM | -1.400673657 | 0.009642995 | Protozoan |
| BMP5 | -1.438101823 | 0.018052056 | Protozoan |
| TNR16 | -2.163413593 | 0.004872747 | Protozoan |
| GLPA | 0.967526586 | 0.036459425 | Viral |
| ITAE | 0.943034385 | 0.034031329 | Viral |
| TNR6 | 0.774998752 | 0.007186927 | Viral |
| NTF4 | 0.723509746 | 0.045457726 | Viral |
| VEGF165, VEGF121 | 0.611098428 | 0.032244582 | Viral |
| PDCD1 | 0.607303639 | 0.036859319 | Viral |
| ICAM1 | 0.60660594 | 0.0087838 | Viral |
| TNFL6 | 0.514592247 | 0.016823701 | Viral |
| TNR11 | -0.526877897 | 0.049053958 | Viral |
| FLT3L | -0.592005413 | 0.01695363 | Viral |
| PD1L2 | -0.594151784 | 0.028296702 | Viral |
| SLAF8 | -0.599029613 | 0.016909382 | Viral |
| LFA3 | -0.638671922 | 0.013864188 | Viral |
| CCR7 | -0.666579468 | 0.016945324 | Viral |
| CCL14 | -0.689910125 | 8.17E-05 | Viral |
| IL3RA | -0.703193516 | 0.013758188 | Viral |
| CCL1 | -0.70779339 | 0.006715265 | Viral |
| IL17C | -0.728443444 | 0.043372357 | Viral |
| IL5 | -0.810554299 | 0.015079474 | Viral |

**Supplemental Table 5**

Detail information on pathway enrichment analysis results for significantly changed proteins at convalescence.

(An Excel spreadsheet has been uploaded separately)

**Supplemental Table 6:** Upregulated proteins observed in each disease at Acute infection compared to convalescence**.**

| **Protein** | **logFC** | **pvalue** | **Disease** |
| --- | --- | --- | --- |
| ENTP1 | 1.80998734 | 0.00555227 | BKV |
| CSF1 | 0.97853913 | 0.00325402 | BKV |
| OSTP | 0.9472769 | 0.00515337 | BKV |
| 5NTD | 0.89100025 | 0.00866977 | BKV |
| TGFB1,2,3 | 0.73905892 | 0.00541496 | BKV |
| IL1R1 | 0.56269007 | 0.02728895 | BKV |
| FLT3 | 0.51891305 | 0.04023099 | BKV |
| KSYK | -0.657806 | 0.00315604 | BKV |
| CXCL7 | -0.6742212 | 0.01482864 | BKV |
| I36RA | -0.7373576 | 0.01228383 | BKV |
| CXCL6 | -0.7463889 | 0.03394644 | BKV |
| CD6 | -0.8105489 | 0.03905799 | BKV |
| IL9 | -1.0195704 | 0.04863707 | BKV |
| CTLA4 | 1.30138129 | 0.0207635 | CMV |
| VCAM1 | 0.98821765 | 0.00512255 | CMV |
| CD37 | 0.65186775 | 0.03178596 | CMV |
| TGFB1 | 0.62662988 | 0.04608933 | CMV |
| CSF1 | -0.6024023 | 0.03979203 | CMV |
| LYAM2 | -0.6130072 | 0.03941039 | CMV |
| CXL10 | -0.671552 | 0.00288201 | CMV |
| PD1L1 | -0.6798498 | 0.03281825 | CMV |
| CCL17 | -0.7048082 | 0.00053048 | CMV |
| CD9 | -0.6069998 | 0.02259414 | DEV |
| LIF | -0.6552629 | 0.00562469 | DEV |
| ITA2B | -1.1533129 | 0.01248147 | DEV |
| IL9 | 1.27808498 | 0.01412703 | WNV |
| CXCR5 | 1.18589206 | 0.00913466 | WNV |
| IL5 | 1.13437107 | 0.04060337 | WNV |
| IGF1R | 0.95176779 | 0.02470622 | WNV |
| S100A8/9 | 1.7999848 | 0.00220385 | MLA |
| I17RA | 1.28123504 | 0.01582274 | MLA |
| CCR7 | 0.95589422 | 0.02991599 | MLA |
| CSF1R | 0.80547121 | 0.04451425 | MLA |
| CO3 | 0.74390467 | 0.01774319 | MLA |
| DPP4 | 0.50826752 | 0.03457005 | MLA |
| IL17B | -0.725657 | 0.02842834 | MLA |
| BTLA | 1.22342942 | 0.00712126 | LD |
| CSF3R | 0.75454939 | 0.01168959 | LD |
| CD276 | 2.51073904 | 0.00503134 | TB |
| S100A8/9 | 1.82014918 | 0.0005977 | TB |
| GP1BA | 1.67454355 | 0.00313466 | TB |
| CD45RB | 1.49075485 | 0.00964628 | TB |
| CDw17 | 1.11992431 | 0.0161911 | TB |
| CD24 | 0.91310269 | 0.004278 | TB |
| CD3E | 0.91278517 | 0.04760427 | TB |
| LFA3 | 0.89792492 | 0.00732052 | TB |
| VEGFA | 0.863838 | 0.00188034 | TB |
| ERBB2 | 0.73198715 | 0.00771296 | TB |
| PERM | -0.8645197 | 0.03992362 | TB |
| C163A | -0.9413538 | 0.03919324 | TB |
| IL34 | -0.9640774 | 0.01953568 | TB |

**Supplemental Table 7.** Detail description of the associated pathways

(This table is provided as a separate Excel spreadsheet)
